# Current status of clinical trial research and application of immune checkpoint inhibitors for non-small cell lung cancer

**DOI:** 10.3389/fonc.2023.1213297

**Published:** 2023-09-01

**Authors:** Fuli Wang, Teng Xia, Zhiqiang Li, Xuzhu Gao, Xinjian Fang

**Affiliations:** ^1^ Department of Oncology, Lianyungang Clinical College Affiliated to Bengbu Medical College, Lianyungang, China; ^2^ Department of Oncology, Gaochun Hospital Afliated to Jiangsu University, Nanjing, China

**Keywords:** non-small cell lung cancer, immune checkpoint inhibitors, CTLA-4, PD-1, PD-L1

## Abstract

Immunotherapy has emerged as a hot topic in the treatment of non-small cell lung cancer (NSCLC) with remarkable success. Compared to chemotherapy patients, the 5-year survival rate for immunotherapy patients is 3-fold higher, approximately 4%–5% versus 15%–16%, respectively. Immunotherapies include chimeric antigen receptor T-cell (CAR-T) therapy, tumor vaccines, immune checkpoint inhibitors, and so forth. Among them, immune checkpoint inhibitors are in the spotlight. Common immune checkpoint inhibitors (ICIs) currently in clinical use include programmed death receptor-1(PD-1)/programmed death ligand-1(PD-L1) and cytotoxic T lymphocyte-associated antigen 4(CTLA-4). This article focuses on monotherapy and combination therapy of CTLA-4 and PD-1/PD-L1 immune checkpoint inhibitors. In particular, the combination therapy of ICIs includes the combination of ICIs and chemotherapy, the combination therapy of dual ICIs, the combination of ICIs and anti-angiogenic drugs, the combination of ICIs and radiotherapy, and the combination of ICIs inhibitors and tumor vaccines and so forth. This article focuses on the combination therapy of ICIs with chemotherapy, the combination therapy of dual ICIs, and the combination therapy of ICIs with anti-angiogenic drugs. The efficacy and safety of ICIs as single agents in NSCLC have been demonstrated in many trials. However, ICIs plus chemotherapy regimens offer significant advantages in the treatment of NSCLC with little to no dramatic increase in toxicity, while combined dual ICIs significantly reduce the adverse effects (AEs) of chemotherapy. ICIs plus anti-angiogenic agents regimen improves anti-tumor activity and safety and is expected to be the new paradigm for the treatment of advanced NSCLC. Despite some limitations, these agents have achieved better overall survival rates. In this article, we review the current status and progress of research on ICIs in NSCLC in recent years, aiming to better guide the individualized treatment of NSCLC patients.

## 1 Introduction

Lung cancer is the second most common cancer worldwide and the leading cause of cancer deaths, accounting for 11.4% of new cancers and 18% of cancer-related deaths in 2020 (1). According to statistics, NSCLC accounts for 80%–90% of all lung cancer diagnoses (2). Surgical resection is the main treatment modality for early-stage NSCLC; however, the prognosis and 5-year survival rate of patients after surgery remain unsatisfactory. In addition, approximately two-thirds of patients have already developed local or distant metastases at the time of detection and lost the opportunity for surgery (3). NSCLC is characterized by rapid proliferation, which will multiply rapidly during the radiotherapy stage, usually starting to multiply in 3-4 weeks of radiotherapy, and is also the main factor leading to the failure of radiotherapy, so the overall effect of conventional radiotherapy in treating NSCLC is unsatisfactory (4). Platinum-based two-drug chemotherapy is the standard first-line treatment for advanced negative mutation-driven NSCLC. However, the median overall survival (OS) is only 7.9 months, and chemotherapy-related side effects are not well tolerated by many patients (5). At present, numerous clinical studies have shown that immune checkpoint inhibitors (ICIs) are safer and more effective than conventional treatments, such as radiotherapy and chemotherapy. ICIs have better guidance for the clinical treatment of patients with advanced NSCLC.

This article reviews the mechanism of action of programmed death receptor-1 (PD-1)/programmed death ligand-1 (PD-L1) and cytotoxic T lymphocyte-associated antigen 4 (CTLA-4) ICIs, including their clinical applications and related clinical trials, focusing on clinical trials related to ICIs to provide ideas for treatment options for NSCLC patients.

## 2 Mechanisms of ICIs for NSCLC

**Figure 1:**
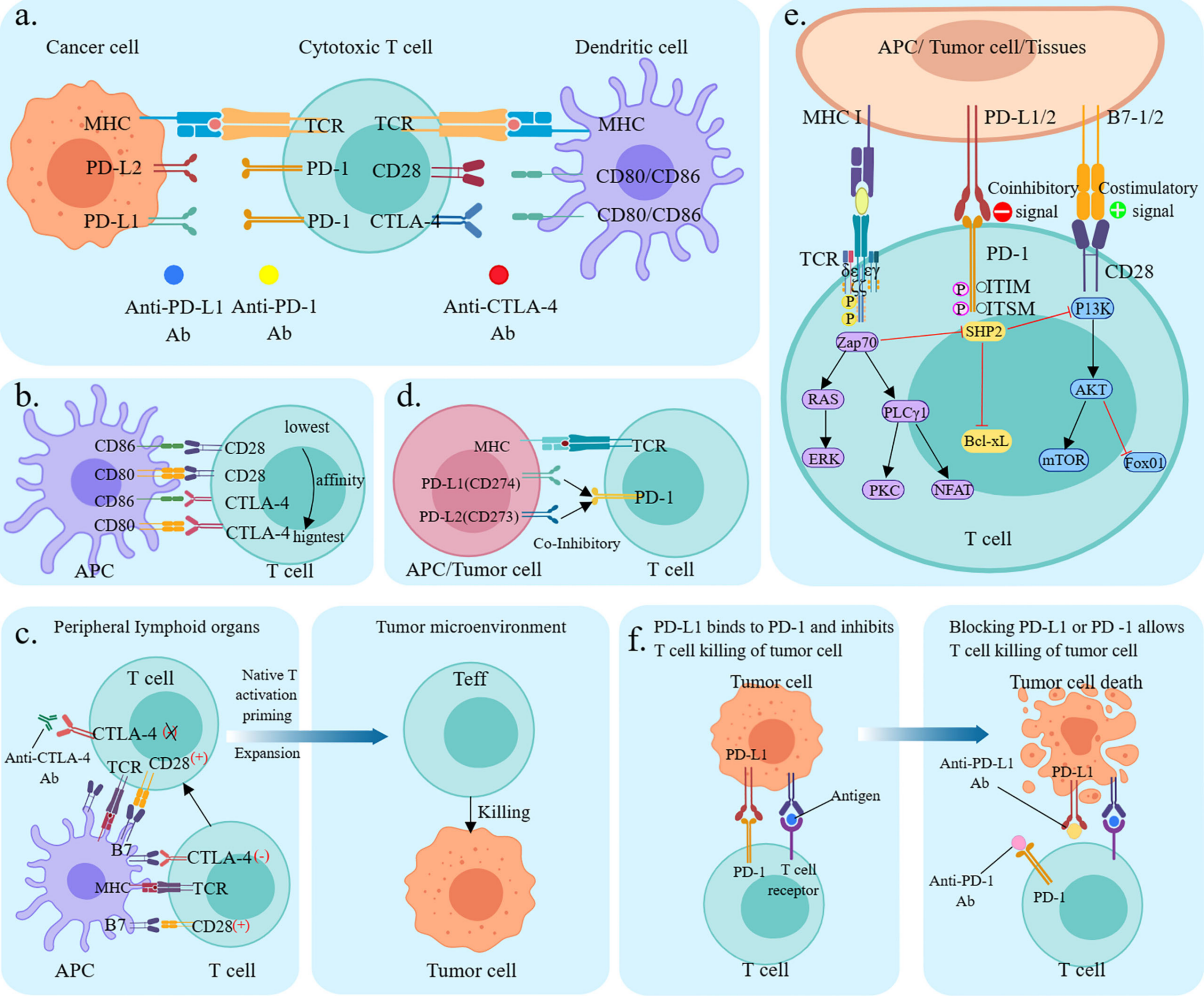
Mechanism of immune checkpoint inhibitors. **(A)** Mechanism of T cell activation and mechanism of action of immune checkpoint inhibitors. TCR presents antigen in MHC molecules on APC, CD80/CD86 ligands on APC co-stimulate interaction with CD28 receptors on T cells. cTLA-4 competitively binds CD80/CD86, PD-1 binds to its ligand PD-L1/PD-L2. Anti-CTLA-4, anti-PD-1, and anti-PD-L1 antibodies block the corresponding immune checkpoint pathways and restore T-cell activity; **(B)** Relative ligand affinity of CTLA-4 and CD28. **(C)** Classical CTLA-4 immunosuppressive mechanism of action; **(D)** PD-L1, PD-L2 competitively combined with PD-1; **(E)** Signaling pathways **(F) **Mechanism of action of PD-1/PDL1 immunosuppressants.

According to immunology, in tumorigenesis and regression, the body resists tumorigenesis through acquired immunity, while tumor cells evade recognition and attack by the body’s immune system through various mechanisms, which then grow and metastasize free of immune killing effects. The immune response begins with antigen uptake, processing, and presentation by antigen-presenting cells (APC), which bind to the major histocompatibility complex molecules through the APC to the T cell surface receptors. The T cell cluster of differentiation (CD)28 receptor then binds to the CD80/CD86 ligand on the APC, and the two signals together activate the T cell (6). Ligands of immune checkpoints bind to receptors, thereby inhibiting the activation of CTLs, which is one of the key causes of tumor immune escape (7). Tumor cells can upregulate the molecular expression of cell surface immune checkpoints and use the immune checkpoint pathway to evade the host immune system, thereby suppressing immune cell function (**Figure 1A**) (8).

### 2.1 Anti-CTLA-4 antibody

CTLA-4 is a suppressor receptor expressed only by T cells and is used to inhibit T cell activity. Although CTLA-4 and CD28 are homologous analogs, they produce different effects. CD28 exerts positive regulation of the immune response, while CTLA-4 exerts negative regulation of the immune response. CTLA-4 has a higher affinity for CD80/CD86 than CD28, thus CTLA-4 pre-emptively binds to CD80/CD86 through competitive action. In addition, CTLA-4 can downregulate CD80/CD86 expression on APC or remove it through cellular cytokinesis, blocking the B7-CD28 signaling pathway in T-cell activation by competing with CD28, and thus blocking T-cell activation (**Figure 1B**) **(** 9). CTLA-4 plays a negative regulatory role in T cell activation and activation of the immune response, and anti-CTLA-4 antibodies block inhibitory signals, induce T cell activation and proliferation, and restore their function. In addition, CTLA-4 induces the development and function of regulatory T cells (Treg), and CTLA-4 deficiency impairs Treg suppressor function *in vivo* and *in vitro* (**Figure 1C**) (10).

### 2.2 Anti-PD-1/PD-L1 antibody

PD-1 (also known as CD279) is an important immune checkpoint protein, mainly expressed in activated T cells and is associated with specific ligands PD-L1 (B7-H1, CD274) and PD-L2 (B7-H2, CD273) (11). Both can competitively bind to the PD-1 interaction. The PD-L2/PD-1 interaction has a higher affinity for cancer cells than the differentiated PD-L1/PD-1 interaction and activation; however, the tumor expression and activity of differentiated PD-L2 in regulatory cancer cells is much less than that of PD-L1 (12). PD-L1 is induced to be expressed by immune cells and epithelial cells, and PD-L2 is induced to be expressed by APC. Physiologically, PD-1 interacts with PD-L1 and PD-L2 on the surface of APC to inhibit T-cell overactivation and maintain immune homeostasis (**Figure 1D**). When tumor cells expressing PD-L2 bind to PD-1 (CD279) on T cells for synergistic signaling, it induces dephosphorylation of binding protein tyrosine phosphatases and affects downstream signaling pathways such as PI3K/Akt, Ras/ERK, PLCγ, and VAV, leading to immunosuppression and cancer progression by inhibiting T cell activation, proliferation, survival, and cytolytic functions in the tumor microenvironment (**Figure 1E**) (11). PD-1/PD-L1 monoclonal antibody disrupts tumor immune tolerance by specifically blocking PD-1/PD-L1 interaction, restoring the killing function of tumor-specific T cells and achieving tumor clearance (**Figure 1F**) **(** 13).

## 3 Research progress of ICIs

### 3.1 CTLA-4 ICIs

Ipilimumab is a CTLA-4 immunoglobulin G1 (IgG1) monoclonal antibody, which enhances T cell activation and proliferation for anti-tumor effects. A randomized phase II study showed that ipilimumab in combination with chemotherapy improved outcomes more significantly than chemotherapy alone in patients with driver-negative squamous NSCLC (sq-NSCLC) (14). In a subsequent phase III trial, patients with advanced sq-NSCLC who had not received chemotherapy were randomized to either the ipilimumab +paclitaxel+ carboplatin(I+CP)or placebo in combination with chemotherapy groups and found no significant difference in OS between the two groups (median OS:13.4 months vs12.4 months), with a median progression-free survival (PFS) of 5.6 months in both groups. The results suggest that I+CP did not improve OS in patients with advanced sq-NSCLC and resulted in a higher incidence of treatment-related adverse events (15). It has been hypothesized that Ipilimumab, which stimulates early T-cell activation in the lymphoid region, may not produce a sufficiently strong anti-tumor response in SCLC if there is no corresponding effector T-cell stimulation in the local tumor microenvironment. This explanation may also apply to sq-NSCLC (15) Nivolumab, a fully human anti-PD-1 antibody, and ipilimumab, a fully human anti-CTLA4 antibody, are ICIs with distinct but complementary mechanisms of action. Ipilimumab induces T-cell proliferation and *de-novo* anti-tumor T-cell responses, including in memory T cells, whereas nivolumab restores the function of existing anti-tumor T cells (16–18). 2020 FDA approval for Nivolumab+ipilimumab+chemotherapy first line for advanced or relapsed NSCLC.

### 3.2 PD-1 ICIs

#### 3.2.1 PD-1 ICIs monotherapy

Pembrolizumab is a monoclonal antibody that targets PD-1 and binds to the PD-L1 receptor, blocking its interaction with PD-L1 and PD-L2. KEYNOTE-001 was the first phase Ib study to evaluate pembrolizumab in patients with advanced NSCLC, showing an objective response rate (ORR) of 27%, median OS of 22.1 months, and PFS of 6.2 months. Among patients with PD-L1 tumor proportion score (TPS) ≥50%, ORR was 45.2% (19). Based on the findings in KEYNOTE-001, KEYNOTE-024 further investigated the efficacy of first-line pembrolizumab monotherapy in advanced NSCLC patients with PD-L1 TPS ≥50% and compared it with chemotherapy (20). The study found that the pembrolizumab group had significantly better PFS and ORR than the chemotherapy group; however, OS had not been reached. Based on the KEYNOTE-001/024 study, the US Food and Drug Administration (FDA) announced the approval of pembrolizumab as the first-line treatment for patients with advanced NSCLC with high PD-L1 expression (21). To further expand the population for first-line immunotherapy, the KEYNOTE-042 study was created. This study demonstrated that immunotherapy was more effective than chemotherapy in patients with PD-L1 TPS ≥1%, with lower rates of side effects and an extended OS of nearly 8 months. Furthermore, the study included a Chinese population for the first time, thus allowing the results to be more relevant to Chinese patients as well (22). Based on the results of the KEYNOTE-042 study, The National Medical Products Administration approved pembrolizumab as a single-agent first-line treatment for advanced NSCLC with PD-L1 TPS ≥1% in September 2019. The results of the KEYNOTE024 and KEYNOTE042 studies showed that people with high PD-L1 expression benefitted more from immune monotherapy (20, 22).

Nivolumab, an IgG4 monoclonal antibody that binds to the PD-1 receptor, blocks the interaction of PD-1 with PD-L1 and PD-L2 and relieves PD-1 pathway-mediated suppression of the immune response. It is the first FDA-approved humanized IgG4-type monoclonal antibody against PD-1 as the second-line treatment for advanced or metastatic NSCLC, with a high safety profile and durable efficacy (23, 24). The CheckMate017 study showed that in patients with advanced sq-NSCLC cancer, the (median OS: 9.2 months vs 6.0 months), (median PFS: 3.5 months vs 2.8 months) and response rates were significantly better in the nivolumab group than in the docetaxel group, regardless of PD-L1 expression levels (25). Check Mate-063 results showed an ORR of 14.5% and a 1-year OS rate of 39%, demonstrating the significant benefits of nivolumab in relapsed refractory sq-NSCLC (26). Subsequent Check Mate-012 results showed a 23% ORR and 74% 1-year OS rate for nivolumab monotherapy in advanced NSCLC, confirming a significant prolongation of the duration of response (DoR) for nivolumab monotherapy in first-line treatment of advanced NSCLC (24). In the CheckMate 078 and 057 trials, nivolumab had a significant improvement in patient survival and a significantly lower incidence of AEs compared to docetaxel, and the benefit was also more pronounced in patients with low PD-L1 expression (27, 28).

#### 3.2.2 PD-1 ICIs in combination with chemotherapy

Tislelizumab is a humanized IgG4 monoclonal antibody with high affinity and specificity for PD-1, which rarely binds to FcγR on macrophages, thus eliminating antibody-dependent phagocytosis, T-cell clearance mechanisms, and potential resistance to anti-PD-1 therapy (29, 30). A study revealed that tislelizumab was well tolerated in patients with advanced solid tumors, regardless of PD-1 expression, and anti-tumor activity was observed in NSCLC (31). RATIONALE304, a phase III clinical trial in nonsquamous NSCLC (nsq-NSCLC), showed that PFS was significantly longer in stage IIIB or IV nsq-NSCLC patients treated with tislelizumab + platinum + pemetrexed(T+PP) as compared to platinum + pemetrexed (PP)(median PFS:9.7 months vs 7.6 months). Furthermore, the main adverse effect (AE) of this regimen is decreased neutrophil count. In addition, the combination therapy had a higher response rate and longer response time, and the best PFS benefit was observed in patients with ≥50% PD-L1 expression (32). RATIONALE 307 is one of the first phase 3 trials of a PD-1 inhibitor in combination with chemotherapy for sq-NSCLC.Tislelizumab+carboplatin+nab-paclitaxel/paclitaxel (T+CnP/T+CP) dramatically improved PFS and ORR and provided evidence of stable safety/tolerability compared to carboplatin+nab-paclitaxel/paclitaxel(CnP/CP). The study also fully demonstrates the clinical benefit of tislelizumab in combination with chemotherapy as a first-line treatment for sq-NSCLC (33).

Sintilimab is a potent and selective anti-PD-1 antibody that inhibits the interaction between PD-1 and its ligands. Compared to nivolumab and pembrolizumab, sintilimab has a different binding epitope and greater PD-1 binding affinity (34). Platinum and gemcitabine (GP) are the most common inter-standard chemotherapy regimens for sq-NSCLC in Asia. ORIENT12 is the first study to use GP as a backbone combination to assess the benefit of adding an anti-PD-1 antibody to first-line sq-NSCLC chemotherapy in Asia (35). The results showed that the addition of sintilimab+GP (S+GP) standard chemotherapy significantly prolonged PFS in previously untreated patients with advanced or metastatic sq-NSCLC, and the greatest benefit was observed in the subgroup with PD-L1TPS >50%. Furthermore, this regimen could be used as first-line treatment for locally advanced or metastatic sq-NSCLC. ORIENT11 showed that in patients with previously untreated, locally advanced, or metastatic nsq-NSCLC, the addition of sintilimab+pemetrexed+platinum (S+PP) significantly prolonged PFS (median PFS:8.9 months vs 5.0 months) with a manageable safety profile compared to chemotherapy alone. Thus, the combination regimen may provide a new treatment option for this patient population (36).

Camrelizumab (SHR-1210), a humanized Ig G4-k monoclonal antibody against PD-1, exhibits anti-tumor activity and tolerability in lung cancer (37). In the phase 3 CameL trial, camrelizumab + pemetrexed + platinum (C+PP) significantly prolonged PFS compared to chemotherapy (PFS:11.3 months vs 8.3 months). The main AEs of this regimen are decreased white blood cell and neutrophil counts and anemia. The regimen is identified as the standard first-line therapy for Chinese patients with advanced nsq-NSCLC without EGFR mutations or ALK translocations (38). In the CAMEL-SQ study, first-line camrelizumab + carboplatin+ paclitaxel (C+CP) showed stable and durable clinical benefit in patients with advanced sq-NSCLC (median PFS:8.5 months vs 4.9 months) (39). Although the OS had not been reached, the survival benefit was consistent across all PD-L1 TPS subgroups, with an ORR of 64.8% versus 36.7%, DoR of 13.1 versus 4.4 months, and manageable adverse events. These findings support the efficacy of C+CP as the standard first-line treatment regimen for sq-NSCLC.

### 3.3 PD-L1 inhibitor

#### 3.3.1 PD-L1 ICIs monotherapy

Durvalumab is a humanized anti-PD-L1 protein monoclonal antibody that blocks the binding of PD-Ll to PD-1 and CD80. It recognizes and clears tumor cells and can be used as the first-line treatment for unresectable III NSCLC that has not progressed after concurrent radiotherapy or chemotherapy and for progressing SCLC (40, 41). A phase III study [NCT02125461] showed a higher PFS in the durvalumab group than in the placebo group (PFS:16.8 months vs 5.6 months) (42). In addition, the ORR was higher in the durvalumab group than in the placebo group (28.4% vs 16.0%), with a DoR of 18 months. Similarly, the median time to death or distant metastasis was longer in the durvalumab group compared to the placebo group (23.2 months vs 14.6 months). In the phase III ARCTIC study (NCT02352948), 476 patients with advanced NSCLC received durvalumab as a consolidation therapy after chemoradiotherapy (43). The study found that patients treated with durvalumab had a longer median PFS benefit irrespective of PD-Ll expression levels. In light of these findings, in February 2018, the US FDA approved durvalumab for patients with NSCLC whose disease has not progressed after locally advanced chemoradiotherapy and who are inoperable.

Avelumab is an IgG1-type monoclonal antibody, which also has antibody-dependent cell-mediated cytotoxic effects compared to other PD-L1 inhibitors, causing direct lysis of tumor cells (44). Avelumab has a controlled safety profile and promising clinical activity in a population of patients with progressive, platinum-treated, metastatic, or recurrent NSCLC. Responses occurred in both squamous and non-squamous tumors, regardless of PD-L1 expression status. These findings support the therapeutic benefit of anti-PD-L1 antibodies in previously treated NSCLC patients. In addition, these results demonstrating the efficacy of avelumab provide a rationale for ongoing Phase 3 trials in the second-line NSCLC population and highlight the potential benefit of immunotherapy for patients with this difficult-to-treat disease (45).

#### 3.3.2 PD-L1 ICIs combined with chemotherapy

Atezolizumab is an engineered humanized monoclonal anti-PD-L1 antibody that inhibits the binding of PD-L1 to PD-1 and B7.1 (also known as CD80), thereby restoring anti-cancer immunity (46). IMpower130 is the first to demonstrate the benefit of PD-L1 inhibitors in combination with chemotherapy for the first-line treatment of advanced NSCLC. The results of the study showed that atezolizumab+carboplatin+nab-paclitaxel(A+CnP) for first-line treatment of patients with EGFR/ALK wild-type nsq-NSCLC showed a better benefit in both OS (median OS: 18.6 months vs 13.9 months) and PFS (median PFS:7.0 months vs 5.5 months) compared to chemotherapy, with no new occurrence of AEs (47). In light of these findings, A+CnP was approved by the US FDA for the first-line treatment of metastatic nsq-NSCLC without EGFR/ALK mutations. In the phase III clinical trial IMpower131, which also compared the efficacy of immunotherapy plus chemotherapy with chemotherapy alone in advanced sq-NSCLC, there was an improvement in PFS in the A+CnP group compared with CnP group (PFS:6.3 months vs 5.6 months), with no difference in OS (48). The IMpower132 study focused on the efficacy of atezolizumab+ pemetrexed + platinum(A+PP) versus chemotherapy alone in patients with advanced nsqNSCLC and showed that atezolizumab in combination with chemotherapy improved PFS (PFS: 7.6 months vs 5.2 months), regardless of PD-L1 expression (49).

Sugemalimab (formerly CS1001) is an immunoglobulin G4 (IgG4, s228p) monoclonal antibody targeting PD-L1. Sugemalimab retains binding affinity to Fcγ receptor I and thus can effectively induce antibody-dependent cellular phagocytosis through cross-linking of PD-L1-positive tumor cells with macrophages prevalent in the tumor microenvironment and may further enhance tumor antigen presentation (50). In the GESTONE-302 trial, which investigated the PD-L1 inhibitor sugemalimab in combination with platinum-based chemotherapy (S+P) in NSCLC, sugemalimab combined with chemotherapy improved PFS compared with placebo combined with chemotherapy (median PFS:9.0 months vs 4.9 months), with a more prominent benefit, especially in the sq-NSCLC subgroup. Analysis of the subgroups indicated that these benefits remained unchanged, regardless of PD-L1 expression and NSCLC subtype. Their results confirmed that sugemalimab combined with platinum-based chemotherapy showed measurable improvements in PFS in NSCLC patients and could be a new first-line treatment option for NSCLC (51). An interim analysis of the phase 3 trial GESTONE-301 exhibited a significant and clinically meaningful improvement in PFS after concurrent or sequential chemoradiotherapy combined with sugemalimab (S+C)group compared with the placebo group. The results suggested that sugemalimab is an effective consolidation therapy for patients with locally advanced, unresectable stage III NSCLC without disease progression after chemoradiotherapy (52).

### 3.4 Combined treatment with dual ICIs

**Figure 2:**
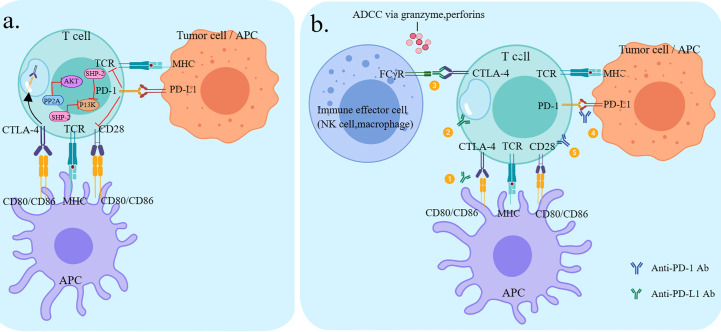
Checkpoint pathways of CTLA-4 and PD-1 and potential mechanisms of action of antibodies. **(A)** CTLA-4 and PD-1 pathways negatively regulate T-cell activation **(B)** ① Anti-CTLA-4 restores T-cell activation by inhibiting the interaction between CTLA-4 and CD80/CD86 on the APC.② Anti-CTLA-4 inhibits CD28 ligand CD80/86-mediated endocytosis *via* CTL A-4-mediated endocytosis.③Anti-CTLA-4 lgG1 antibody-ipilimumab, through its Fc segment, binds to FcyR° on immune effector cells (NK cells, monocytes/macrophages), resulting in ADCC effects and subsequent depletion of highly expressed CTLA-4 T cell subsets (e.g. Treg).④ Anti-PD-1 restores T-cell activation by inhibiting the interaction between PD-1 and PD-L1 on T cells (PD-L1 can be expressed by tumor cells and various immune cells.⑤ Anti-PD-1 restores Tcel activation through the interaction between PD-1 and CD28, which is the point of convergence between the two pathways.

PD-1 and CTLA-4 are both immune checkpoint molecules but have very different mechanisms of action, negatively regulating the activation of T cells in the immune response at different stages. CTLA-4 prevents T cell activation and effector functions during the initial T cell activation phase, whereas PD-1 acts on activated T cells at a later stage of the immune response, inhibiting the degree of T cell activation and cytotoxicity (**Figure 2A**) **(** 13). Clinical research has shown that the combination of PD-1/PD-L1 inhibitors and CTLA-4 inhibitors resulted in enhanced effector T-cell action and attenuated suppressor T-cell action, resulting in stronger anti-tumor effects than single-agent ICIs (**Figure 2B**) **(** 53).

The CheckMate012 study was a trial of dual ICIs regimen, using nivolumab and ipilimumab as the first-line treatment for patients with NSCLC. The regimen showed excellent efficacy with a manageable safety profile. Patients with high PD-L expression benefitted more from combination therapy. This investigation was the first to support that dual immunotherapy can enhance the benefit of first-line treatment in NSCLC (54). The CheckMate 227 trial further confirmed that the dual ICIs arm (nivolumab plus ipilimumab, N+I) had a prolonged PFS (median PFS:7.2 months vs 5.4 months) and a more prominent improvement in ORR (45.3% vs 26.9%) compared to the chemotherapy alone arm, regardless of PD-L1 status. The results of this project were assessed by the Lung Cancer Symptom Scale and the European Five-Dimensional Health Scale, and patient-reported outcomes showed multiple symptoms and quality-of-life improvements compared to chemotherapy (55). The results of the CheckMate 9LA study were consistent with that of CheckMate 227. Compared to chemotherapy alone, regardless of tumor histology or PD-L1 expression, nivolumab plus ipilimumab in combination with two cycles of chemotherapy significantly improved survival. Efficacy and safety data support nivolumab plus ipilimumab combination chemotherapy has a favorable risk-benefit profile as first-line therapy for patients with advanced NSCLC (56). The main objective of the phase III clinical trial MYSTIC study was to explore the efficacy and safety of durvalumab plus tremelimumab versus conventional chemotherapy in the first-line treatment of advanced NSCLC and to explore its associated biomarkers. At tumor mutation burden (TMB) ≥16 mut/Mb, the OS of dual immune combination versus chemotherapy was 16.5 months and 10.5 months, with significant differences. In patients with low TMB, immunotherapy did not result in favorable OS, showing the importance of appropriate biomarkers (57). A phase 1b investigation demonstrated that, regardless of PD-l expression levels, the combination of durvalumab and tremelimumab showed good anti-tumor effects in NSCLC patients (58).

### 3.5 ICIs in combination with anti-angiogenic drugs

ICIs combined with anti-angiogenic drugs are based on the following theories. First, tumor angiogenesis inhibits the tumor microenvironment; vascular endothelial growth factor (VEGF) plays a key role in tumor angiogenesis and immunosuppression at different levels by binding to VEGF receptors 1-3 and neuropilin (59). Dendritic cells (DCs) play a central role in T cell initiation and activation. However, VEGF can inhibit the differentiation, maturation, and antigen presentation of DCs (60). In addition, VEGF can drive the suppressive effects on effector T cells by inhibiting the differentiation of progenitor cells to CD8+ T cells, reducing the proliferation and cytotoxic effects of CD8+ T cells, increasing the exhaustion of CD8+ T cells, promoting the polarization of tumor-associated macrophages (TAMs) to M2 type and recruiting immunosuppressive cells (such as Tregs, MDSCs and M2-like TAMs) to exert immunosuppressive effects (61). Target VEGF can diminish the expression of adhesion molecules on the endothelium of tumor vessels and decrease the ability of immune cells to adhere to and cross the vessel wall, thus preventing immune cells from entering the tumor (62). Second, the tumor immune microenvironment promotes tumor angiogenesis; neuropilin-1 can be transferred from DCs to T cells during the interaction between T cells and DCs, and the transferred neuropilin-1 can effectively bind VEGF secreted by DCs to boost tumor angiogenesis. Moreover, DCs and M2-like TAMs can promote angiogenesis by secreting the pro-angiogenic factor VEGF (63).

**Figure 3:**
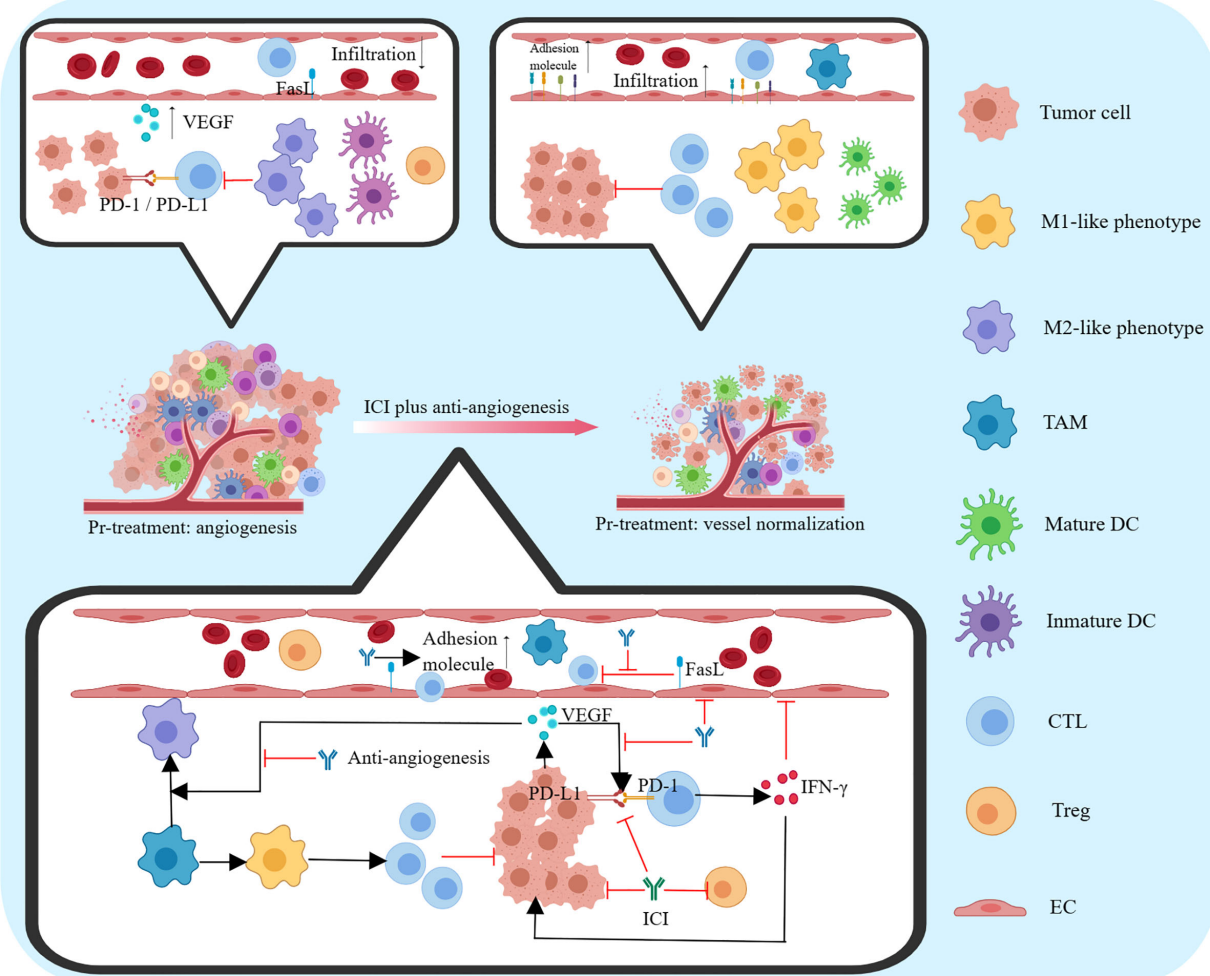
Mechanism of action of ICIs in combination with anti-angiogenic agents.

Mechanism of action of anti-angiogenic drugs: 1) Immune response is stimulated by increasing CD8+ T lymphocyte infiltration into the tumor (64); 2) immune signaling is suppressed by inhibiting T regulatory cell proliferation and DC maturation, and PD-1 expression in infiltrating tumor T lymphocytes exerts a regulatory effect; 3) TAMs are induced to polarize into an immune-supporting M1-like phenotype, and the expression of immune checkpoint molecules, such as PD-L1 and CTLA-4, on the surface of immunosuppressive cells and the secretion of immunosuppressive factors, such as VEGF, transforming growth factor β and interleukin 10, is reduced, thereby restoring the activation and function of immune cells (65); and 4) reducing vascular pressure, improving tissue hypoxia, inviting vascular normalization and relieving immunosuppression by depressing the permeability of tumor vessels (66). On the other hand, ICIs can activate CD8+ T lymphocytes and Th1 cells to secrete anti-tumor cytokines such as interferon γ and tumor necrosis factor, which can regulate the immune microenvironment while exerting anti-angiogenic and vascular normalization effects (67, 68). During anti-angiogenic drug treatment, immunotherapy can be coupled with immunotherapy to enhance the transport of immunotherapeutic drugs and immune cells, strengthen the infiltration of immune cells into tumor tissues and activate the positive regulation of the body’s immune function to achieve reinforcement of the anti-tumor effect. Therefore, the combination of anti-angiogenic and immunotherapy can theoretically produce a synergistic anti-tumor effect (**Figure 3**).

IMpower150, a representative study of ICIs-combined anti-angiogenesis, is the first randomized phase III clinical trial to demonstrate the benefit of ICIs in patients with EGFR mutations. The results showed that the atezolizumab+bevacizumab+ carboplatin + paclitaxel (ABCP) arm prolonged PFS and OS in first-line nsq-NSCLC patients compared to the bevacizumab+ carboplatin + paclitaxel (BCP) arm, including the EGFR mutation and ALK translocation populations. The exploratory analysis in the EGFR mutation population suggested that patients with EGFR-sensitive mutations or NSCLC treated with tyrosine kinase inhibitors can benefit from the ABCP regimen, adding a new treatment option for this patient population. Following this study, in December 2018, the US FDA approved, atezolizumab+bevacizumab+paclitaxel+carboplatin for the front-line treatment of EGFR/ALK-negative metastatic nsq-NSCLC, regardless of PD-L1 expression status (69). ONO-4538-52/TASUKI-52, a randomized, double-blind phase III clinical trial, evaluated nivolumab+bevacizumab+carboplatin+paclitaxel (NBCP) as a first-line treatment for nsq-NSCLC (70). The outcomes showed that the median PFS was significantly longer in the ABCP group than in the placebo group (median PFS:12.1 months vs 8.1 months), and prolonged PFS was observed in all patients with PD-L1 expression levels, with an ORR of 61.5% and 50.5%, respectively. In addition, the incidence of grade ≥3 treatment-related AEs was comparable in both groups. This regimen can be considered a new viable treatment strategy for patients with primary nsq-NSCLC. In the JVDF study (NCT02443324), 26 patients with advanced NSCLC were enrolled and were administrated ramucirumab+pembrolizumab (P+R) as first-line treatment. By the time of data cut-off, the overall ORR was 42.3%, the disease control rate (DCR) was 84.6%, the median PFS was 9.3 months, OS was not reached and the overall safety profile was excellent, with stratified analysis showing better efficacy in those with high PD-L1 expression than in those with low PD-L1 expression. This study revealed the clinical benefits of anti-angiogenic combination ICIs (71). A phase Ib/II clinical trial (NCT02501096), which included 21 patients with advanced NSCLC who received lenvatinib+pembrolizumab, showed an overall ORR of 33.3%, DCR of 80.9%, median PFS of 7.4 months and overall safety control. On account of these findings, a phase III clinical trial (NCT03829319) was initiated. Data from the first part of LEAP-006 suggested that the effectiveness of pembrolizumab+chemotherapy in combination with lenvatinib in patients with advanced NSCLC as the primary treatment is definite. Furthermore, data from 13 validated analyses showed that the ORR of this combination mode was xx. The second part of the randomized study is currently underway, and we look forward to the publication of the related data (72). Preliminary results from the phase I study (NCT03628521) using sintilimab+anlotinib (S+A) in 22 patients with advanced NSCLC showed that the combination therapy was well tolerated by all patients, with an incidence of grade ≥3, treatment-related adverse events of 31.8%, an ORR of 77.3% and a DCR of 100%. A subgroup analysis according to patients’ PD-L1 expression and TMB at baseline showed a consistent benefit of combination therapy in all subgroups. Although PFS was immature at the time of data cut-off, the regimen demonstrated good anti-tumor activity (73). A phase II study (NCT04239443) explored the efficacy and safety of apatinib+camrelizumab as a second-line and beyond-treatment option for advanced NSCLC. The results showed that among 91 evaluable subjects with nsq-NSCLC, ORR was 30.8%, DCR was 82.4%, median PFS was 5.9 months and OS was not achieved, with stratified analysis showing better clinical outcomes observed in patients with bTMB-high

## 4 Conclusion and prospect

With a large number of clinical trials and a growing body of data demonstrating the durable efficacy of ICIs in patients with advanced NSCLC, the clinical use of ICIs is changing the treatment paradigm and landscape for NSCLC. The study of ICIs for NSCLC has been extended to first-line treatment, and PD-1/PD-L1 ICI monotherapy has improved the prognosis of some patients with advanced NSCLC and become a more favorable treatment after molecular targeted therapy (**Table 1**). Combination therapy with ICIs avoids the intolerable AEs caused by chemotherapy. Combination therapy with ICIs and anti-angiogenic drugs has shown high anti-tumor activity and tolerable safety and is expected to become a new paradigm in the treatment of advanced NSCLC (**Tables 2**, **3**). However, despite its excellent efficacy in NSCLC, ICIs have some limitations. First are the immune-related adverse events (irAEs). A growing body of research data suggests that although ICIs improve survival, a significant proportion of patients develop irAEs. Common target organs for irAEs include the skin, gastrointestinal tract, liver, lungs, and endocrine organs. Common irAEs for CTLA-4 ICIs are colitis, pituitary gland inflammation, and rash. Pneumonia, hypothyroidism, and arthralgia are often seen in PD-1/PD-L1 ICI irAEs. However, although these irAEs are elevated, they are generally within control with proper monitoring and management. Moreover, most irAEs are mild to moderate, although severe or life-threatening irAEs do occur, resulting in death in 1%–2% of patients. The current mainstay of treatment for irAEs is dose reduction or drug discontinuation, and for severe toxic reactions, immunotherapy should be permanently terminated. The second limitation is the lack of validated predictive biomarkers of efficacy. Some studies have shown that the expression level of PD-L1, mismatch repair gene expression status, and TMB, among others, have a certain correlation with the efficacy of PD-1/PD-L1 ICIs. Among them, PD-L1 is the most recommended immunotherapy-related oncology marker by the National Comprehensive Cancer Network guidelines. However, its application is limited by temporal dynamics, tumor heterogeneity, and different threshold detection methods. Therefore, PD-L1 expression may not be the best predictive biomarker for efficacy. Therefore, future studies combining multiple other novel biomarkers to individualize the choice of ICIs treatment regimen are warranted. Third is acquired immune resistance. Although ICIs therapies have improved prognostic outcomes for many NSCLC patients, only a few patients have achieved durable responses after treatment with ICIs. We need to tap into novel immune checkpoint molecules as well as explore combination strategies of different ICIs to address drug resistance. The combination of ICIs with topical therapy (mainly radiofrequency ablation, cryoablation, and bronchial artery chemoembolization, etc.) lacks a large number of reliable clinical studies. Although it has been shown that these topical therapies combined with ICIs in the treatment of NSCLC, can improve the survival rate and prolong the survival of patients. However, there is still a lack of sufficient clinical data, and more evidence-based medical data is needed to validate the findings. This is a new direction for the treatment of NSCLC in the future, and it is worthwhile for us to follow the new research in this field. All of the above questions will be the direction of our future exploration and endeavors, guiding us to continue to improve and expand this area of research to ensure that more NSCLC patients can experience significant improvements in both survival time and quality of life.

**Table 1:** Summary of the efficacy and safety of ICIs monotherapy in NSCLC.

Study	Phase	Treatment				Efficacy			Safety		Most common Aes	Reference
		Experimental group			ORR	PFS	OS	DOR	Any AEs	≥G3AEs		
					(%)	(month)	(month)	(month)	(%)	(%)		
PD-1 ICIs monotherapy												
Keynote001	I	pembrolizumab			27	6.2	22.1	12.5	85.1	11.9	fatigue(27.7%),pruritus(14.9%),rash(13.9%),arthralgia(11.9%), hypothyroidism(13.9%), nausea(11.9%)	(19)
Keynote024	III	pembrolizumab			44.8	10.3	–	Not	73.4	26.6	fatigue(10,4%),pyrexia(10.4%), diarrhea(14.3%),	(20)
Keynote042	III	pembrolizumab	PT%	1	27	7.1	16.7	8.3	63	18	Hypothyroidism(11%) pneumonitis(3%)	(22)
				20	33	6.2	17.7	8.3				
				50	39	5.4	20	10.8				
CheckMate017	III	Nivolumab			20	3.5	9.2	Not	58	7	decreased appetite(11%), asthenia(10%), fatigue(16%),	(25)
CheckMate063	II	Nivolumab			Not	–	8.2	Not	75	17	Fatigue(4%),pneumonitis(3%), diarrhea(3%)	(26)
CheckMate057	III	Nivolumab			19	2.3	12.2	17.2	69	10	Fatigue(16%),nausea(12%), decreased appetite(10%), anemia (10%)	(28)
CheckMate078	III	Nivolumab			16.6	2.8	12	Not	9	5	Rash(12%),fatigue(10%)	(27)
PD-L1 ICIs monotherapy												
CT02125461	III	Durvalumab			28.4	16.8	–	18	96.8	29.9	Cough(35.4%), Dyspnea(22.3%), Pneumonitis(33.9%)	(40)
ARCTIC	III	Durvalumab			22	3.8	11.7	9.5	56.5	22	–	(43)
Impower110	III	Atezolizumab			–	8.1	20.2	–	90.2	30.1	Anemia, neutropenia, thrombocytopenia	(74)
EMPOWER- Lung 1	III	Cemiplimab			39	8.2	Not	16.7	43	28	anemia (16%),neutropenia(10%),thrombocytopenia(8%)	(75)
CTLA-4 ICIs monotherapy												
NCT01772004	I	Avelumab			22	3	8.4	–	99	13	infusion-related reaction(21%),fatigue(25%),nausea(13%)	(45)

**Table 2:** Summary of the efficacy and safety of ICIs combination therapy in NSCLC.

Study	Phase	Treatment				Efficacy						Safety		Most common Aes			Reference
		Experimental group			ORR	PFS		OS		DOR		Any AEs	≥G3AEs				
					(%)	(month)		(month)		(month)		(%)	(%)				
PD-1 ICIs Combined with Chemotherapy																	
Rationale304	III	T+PP			57.4	9.7		Not		8.5		20	2	decreased neutrophil count (DeNE,44.6%), anemia (13.5%), thrombocytopenia(19.4%) leukopenia(21.6%)			(32)
Rationale307	III	A:T+CP			72.5	7.6		–		8.2		99.4	85.8	anemia,alopecia,DeNE			(33)
		B:T+CnP			74.8	7.6		–		8.6			83.9				
Orient11	III	S+PP			51.9	8.9		Not		Not		99.6	61.7	anemia (74.1%),DeNE(71.1%),decreased white blood count (DeWBC,67.7%)			(36)
Orient12	III	S+GP			44.7	6.7		Not		6.1		100	86.6	anemia (93.3%),DeNE(83.2%),DeWBC(88.8%), decreased platelet (72.6%)			(35)
Camel	III	C+PP			60.5	11.3		Not		17.6		99.5	69	DeNE,DeWBC, anemia			(38)
Camel SQ	III	C+CP			64.8	8.5		Not		13.1		–	–	DeNE(155%),DeWBC(30%), anemia (10%)			(39)
Keynote189	III	Pembrolizumab+PP			47.6	88		Not		11.2		99.8	67.2	Nausea, anemia, fatigue			(76)
Keynote407	III	pembrolizumab+CP			57.9	6.4		15.9		7.7		98.2	69.8	anemia,alopecia,neutropenia			(77)
PD-L1 ICIs Combined with Chemotherapy																	
Impower130	III	A+CnP			49.2	7		18.6		8.4	99.6		81	neutropenia(32%), anemia (29%),DeNE(12%)			(47)
Impower131	III	A+CnP			49.7	6.3		14.2		7.3	97.9		68	Pneumonitis(3.0%),neutropenia(3.9%), anemia (2.1%)			(48)
Impower132	III	A+PP			47	7.7		17.1		10.1	98.6		54.6	Rash(25.8%),hypothyroidism(8.2%),pneumonitis(6.2%)			(49)
Gestone301	III	S+C			–	9		–		Not	76		9	pneumonia(2%) interstitial lung disease (2%)			(52)
Gestone302	III	S+P			–	9		–		–	99		54	DeNE (33%), anemia (13%), decreased platelet (10%) DeWBC(14%),			(51)
CTLA-4 ICIs Combined with Chemotherapy																	
NCT01285609	III	I+CP			44	5.6		13.4		–	89		53	anemia (12%), diarrhea(7%), thrombocytopenia(7%) neutropenia(14%),			(15)
Combined treatment with dual ICIs																	
CheckMate012	I	Nivolumab 1 mg/kg + Ipilimumab 1mg/kg q6w			33		5.6		Not	Not	73		40	Skin	Eastpointe	endocrine	(54)
														36%	23%	21%	
		Nivolumab 3mg/kg + Ipilimumab 1 mg/kg q6w			38		3.9		–	Not	744		31		23%	21%	
		Nivolumab3 mg/kg + Ipilimumab 1mg/kg q12			47		8.1		–	Not	84		42	39%	24%	11%	
Checkmate227	III	PD-L1	≥1%	N+I	36.4		5.1		17.1	23.2		77.2	35.5	cutaneous (34.0% any grade, 4.2% grade≥3), endocrine (23.8% any grade, 4.2% grade≥3), gastrointestinal (18.2% any grade, 2.4% grade≥3), hepatic (15.8% any grade, 8.2% grade≥3)			(55)
			<1%	N+I	27.3		5.1		17.2	18	75.7		35				
Checkmate9LA	III	Nivolumab 360mg q3w+ Ipilimumab 1mg/kgq6w +Chemotherapy			38		6.7		15.8	13	92		48	hepatic(14.4%), endocrine(25.7%), cutaneous(40.5%), gastrointestinal(23.3%)			(56)
CheckMate568	II	Nivolumab 3mg/kg q2w+ Ipilimumab 1mg/kg q6w			30		4.2		–	Not	80		29	gastrointestinal toxicities (5%) Skin(30%)			(78)
		Nivolumab 360mg q3w+ Ipilimumab 1 mg/kg q6w+ Chemotherapy			47		10.8		19.4	12.7	94		58				
ARCTIC	III	Durvalumab+ Tremelimumab			26		3.5		11.5	12.2	63.3		23.3	–			(43)
ICIs Combined with anti-angiogenic drugs																	
Impower150	III	ABCP			64		8.3		19.2	9	94.4		55	DeNE, neutropenia, hypertension			(69)
TASUKI-52	III	NBCP			61.5		12.1		–	Not	64		56	DeNE, DeWBC, anemia			(70)
JVDF	I	P+R			42.3		9.3		Not	–	84.6		42.3	Rash(26.9%),Fatigue(19.2%) Hypertension(19.2%), Pruritus (15.4%)			(71)
NCT03628521	Ib/II	S+A			77.3		–		Not	–	–		31.8	Hematuria,hyperuricemia,hypertension			(73)

**Table 3:** Summary of efficacy and safety of FAD-approved ICIs for NSCLC.

Study	Phase	Treatment					Efficacy			Safety		Most common Aes			Reference
		Experimental group				ORR	PFS	OS	DOR	Any AEs	≥G3AEs				
						(%)	(month)	(month)	(month)	(%)	(%)				
ICIs monotherapy															
Keynote001	I	pembrolizumab				27	6.2	22.1	12.5	85.1	11.9	fatigue(27.7%),pruritus(14.9%),rash(13.9%),arthralgia(11.9%), hypothyroidism(13.9%), nausea(11.9%)			(19)
Keynote024	III	pembrolizumab				44.8	10.3	–	Not	73.4	26.6	fatigue(10,4%),pyrexia(10.4%), diarrhea(14.3%),			(20)
Keynote042	III	pembrolizumab	PT%	1		27	7.1	16.7	8.3	63	18	Hypothyroidism(11%) pneumonitis(3%)			(22)
				20		33	6.2	17.7	8.3						
				50		39	5.4	20	10.8						
CheckMate017	III	Nivolumab				20	3.5	9.2	Not	58	7	decreased appetite(11%), asthenia(10%), fatigue(16%),			(25)
CheckMate063	II	Nivolumab				Not	–	8.2	Not	75	17	Fatigue(4%),pneumonitis(3%), diarrhea(3%)			(26)
CheckMate057	III	Nivolumab				19	2.3	12.2	17.2	69	10	Fatigue(16%),nausea(12%), decreased appetite(10%), anemia (10%)			(28)
CheckMate078	III	Nivolumab				16.6	2.8	12	Not	9	5	Rash(12%),fatigue(10%)			(27)
CT02125461	III	Durvalumab				28.4	16.8	–	18	96.8	29.9	Cough(35.4%), Dyspnea(22.3%), Pneumonitis(33.9%)			(40)
ARCTIC	III	Durvalumab				22	3.8	11.7	9.5	56.5	22	–			(43)
Impower110	III	Atezolizumab				–	8.1	20.2	–	90.2	30.1	Anemia, neutropenia, thrombocytopenia			(74)
ICIs combination therapy															
Keynote189	III	Pembrolizumab+PP				47.6	88	Not	11.2	99.8	67.2	Nausea, anemia, fatigue			(76)
Keynote407	III	pembrolizumab+CP				57.9	6.4	15.9	7.7	98.2	69.8	anemia,alopecia,neutropenia			(77)
Impower130	III	A+CnP				49.2	7	18.6	8.4	99.6	81	neutropenia(32%), anemia (29%),DeNE(12%)			(47)
Impower131	III	A+CnP				49.7	6.3	14.2	7.3	97.9	68	Pneumonitis(3.0%),neutropenia(3.9%), anemia (2.1%)			(48)
Impower132	III	A+PP				47	7.7	17.1	10.1	98.6	54.6	Rash(25.8%),hypothyroidism(8.2%),pneumonitis(6.2%)			(49)
Impower150	III	ABCP				64	8.3	19.2	9	94.4	55	DeNE, neutropenia, hypertension			(69)
CheckMate012	I	Nivolumab 1 mg/kg + Ipilimumab 1mg/kg q6w				33	5.6	Not	Not	73	40	Skin	Eastpointe	endocrine	(54)
												36%	23%	21%	
		Nivolumab 3mg/kg + Ipilimumab 1 mg/kg q6w				38	3.9	–	Not	744	31		23%	21%	
		Nivolumab3 mg/kg + Ipilimumab 1mg/kg q12				47	8.1	–	Not	84	42	39%	24%	11%	
Checkmate227	III	PD-L1	≥1%		N+I	36.4	5.1	17.1	23.2	77.2	35.5	cutaneous (34.0% any grade, 4.2% grade≥3), endocrine (23.8% any grade, 4.2% grade≥3), gastrointestinal (18.2% any grade, 2.4% grade≥3), hepatic (15.8% any grade, 8.2% grade≥3)			(55)
			<1%		N+I	27.3	5.1	17.2	18	75.7	35				
Checkmate9LA	III	Nivolumab 360mg q3w+ Ipilimumab 1mg/kgq6w +Chemotherapy				38	6.7	15.8	13	92	48	hepatic(14.4%), endocrine(25.7%), cutaneous(40.5%), gastrointestinal(23.3%)			(56)
CheckMate568	II	Nivolumab 3mg/kg q2w+ Ipilimumab 1mg/kg q6w				30	4.2	–	Not	80	29	gastrointestinal toxicities (5%) Skin(30%)			(78)
		Nivolumab 360mg q3w+ Ipilimumab 1 mg/kg q6w+ Chemotherapy				47	10.8	19.4	12.7	94	58				
TASUKI-52	III	NBCP				61.5	12.1	–	Not	64	56	DeNE, DeWBC, anemia			(70)

## Author contributions

Study concept and design: FW and XF. Analysis and interpretation of data: FW and TX. Drafting of the manuscript: FW and TX. Critical revision of the manuscript for important intellectual content: FW, TX, ZL, XF, and XG. Obtained funding: XF. Study supervision: XF and XG. All authors contributed to the article and approved the submitted version.
